# Imaging for thinned perforator flap harvest: current status and future perspectives

**DOI:** 10.1093/burnst/tkab042

**Published:** 2021-12-17

**Authors:** Yi Min Khoong, Xin Huang, Shuchen Gu, Tao Zan

**Affiliations:** Department of Plastic and Reconstructive Surgery, Shanghai Ninth People’s Hospital, Shanghai Jiao Tong University School of Medicine, Shanghai, 200011, China; Department of Plastic and Reconstructive Surgery, Shanghai Ninth People’s Hospital, Shanghai Jiao Tong University School of Medicine, Shanghai, 200011, China; Department of Plastic and Reconstructive Surgery, Shanghai Ninth People’s Hospital, Shanghai Jiao Tong University School of Medicine, Shanghai, 200011, China; Department of Plastic and Reconstructive Surgery, Shanghai Ninth People’s Hospital, Shanghai Jiao Tong University School of Medicine, Shanghai, 200011, China

**Keywords:** Perforator flap, Imaging, Surgical guidance, Preoperative imaging, Intraoperative guidance, Flap perfusion, Flap thinning, Thinned flap

## Abstract

With advances in anatomical knowledge and technology, increased interest has been directed towards reconstruction with enhanced aesthetic and functional outcomes. A myriad of thinned perforator flap harvest approaches have been developed for this purpose; however, concerns about jeopardizing their vascularity remain. To ensure optimum reconstructive outcome without hampering the flap’s microcirculation, it is important to make good use of the existing advanced imaging modalities that can provide clear visualization of perforator branches, particularly in the adipose layer, and an accurate assessment of flap perfusion. Therefore, this review will highlight the imaging modalities that have been utilized for harvesting a thinned perforator flap from these two perspectives, along with future insights into creating both functionally and aesthetically satisfying, yet simultaneously safe, thinned perforator flaps for the best reconstructive outcomes for patients.

Highlights

- Advancement in imaging technologies for thinned perforator flap harvest with improved aesthetic and functional outcomes without hampering the flap’s microcirculation.
- The literature related to the existing imaging tools used to obtain thin perforator flaps through the identification of perforator branches in the subcutaneous tissues and the assessment of tissue perfusion is reviewed.
- The physiological background of perforator flap thinning and organization of the imaging technologies into those for preoperative anatomic evaluation and intraoperative perfusion assessments are highlighted.
- The authors suggest reasonable choices of imaging modalities at the pre- and intra-operative stages for the respective purposes.
- Future insights into the combined use of sophisticated navigation systems using mixed reality and artificial intelligence with the existing imaging modalities are briefly discussed.

## Background

Perforator flaps have become one of the most widely used flaps today in plastic surgery, owing to their vascular reliability and ease of customization. However, the already relatively thin perforator flap remains problematic in defect reconstructions, which necessitate an even thinner flap, and are less susceptible to retraction than full-thickness skin-grafts. Following anatomical knowledge and technical advancement, the perforator flap has been continually improved with regard to its thickness in order to fulfil the MLT principle of ‘matching, large size and thinner thickness’, which we proposed previously [1], for the best possible cosmetic and functional outcomes. Nonetheless, harvesting a thinned perforator flap is not without its downside. For decades, flap thinning has been performed rather blindly, causing detrimental defects of the vascular territory and flap perfusion [2,3]. Later efforts to avoid vasculature injury to the greatest extent possible with a microscope (microdissection) [4–6] remain cumbersome and risky.

Recent emphasis on surgical precision has driven the development of sophisticated imaging devices. Through anatomical delineation of adipocutaneous perforator branches, both naturally existing and intervention-induced anatomical variations are recognized beforehand, avoiding unnecessary painstaking dissection. Vascular injury jeopardizing flap perfusion could also be identified and salvaged promptly. Taken together, imaging technology ensures safe thinned flap harvests without compromising the prime reconstructive principle of ‘replacing like-with-like’.

Comparative studies on the effectiveness of the different existing imaging modalities in thinned perforator flap harvest will be mandatory to establish evidence-based guidelines or algorithms that are currently lacking. In this review, we will first provide a brief overview of the vascular anatomy and haemodynamics changes of thinned perforator flaps, followed by a discussion of current technologies and an outlook on the future development of image-guided thinned perforator flap harvesting.

## Review

### Vascular anatomy and haemodynamics changes of thinned perforator flaps

**Figure 1.**
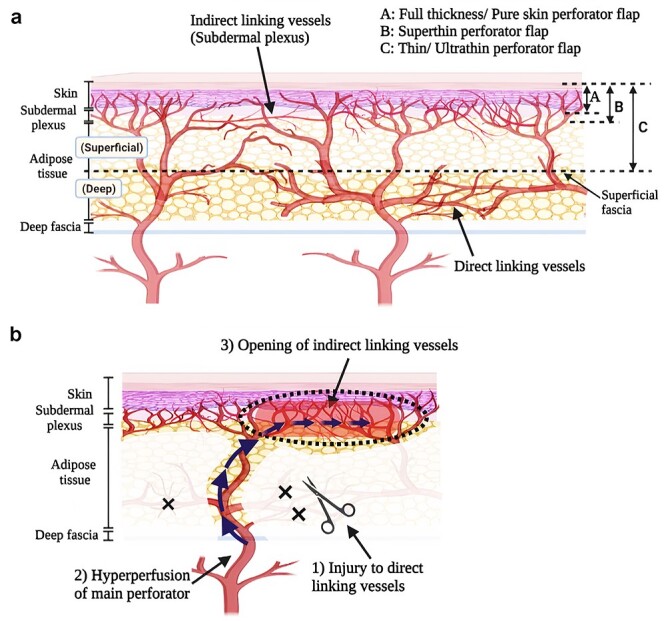
Vascular anatomy and haemodynamic changes of thinned perforator flaps. (**a**) Anatomy and classification of thinned perforator flaps. Adjacent perforasomes are linked through an integrated vasculature that is primarily composed of direct linking vessels organized in the suprafascial and adipose layers and indirect linking vessels constituted in the subdermal plexus. Classifications of thinned perforator flaps: A, full-thickness skin flap or pure skin perforator flap; B, superthin perforator flap (preservation of subdermal plexus); C, thin or ultrathin perforator flap (flap elevation at the superficial fascia plane). (**b**) Underlying physiological mechanism occurring during thinned perforator flap harvest. During thinned flap harvest, numerous direct linking vessels are inevitably injured, leading to activation of the protective mechanism of flap vascularity and hyperperfusion of the main perforator, which consequently results in the opening of the indirect linking vessels constituted by the subdermal plexus. Created with BioRender.com

According to the ‘perforasome theory’ by Saint-Cyr *et al*. [7], perforasomes are linked through an integrated vasculature, primarily composed of the direct linking vessels in the suprafascial and adipose layers and indirect linking vessels constituted in the subdermal plexus (Figure 1a), similar to the true and choke anastomoses described by Taylor *et al*. [8], with ‘protective’ communicating branches between them in cases of vascular injury. The emphasis on subdermal plexus preservation was proposed early in 1967 when the idea of the thinned defatted flap was first introduced [9] and has since been widely adopted in various thinned flaps. Its significance was later proven on perforator flaps through demonstration of the recurrent flow from the subdermal plexus in capturing the adjacent perforasome to maintain flap perfusion [7,10].

According to the perforasome [7] theory, maximizing the recruitment of linking vessels is instrumental in flap perfusion. However, numerous direct linking vessels are inevitably injured during the harvest process. Owing to the naturally built-in protective mechanism of flap vascularity, flap thinning potentially incites hyperperfusion of the main perforator, consequently opening up the indirect linking vessels to maintain flap perfusion [7,8] (Figure 1b). In 2011, Narushima [11,12] challenged the widely accepted notion of subdermal plexus preservation through successful pure skin perforator flap elevation; however its comprehensive anatomical vascularity requires further investigation.

Due to the absence of standard nomenclature for thinned perforator flaps, many terms have been used interchangeably to reflect the extent of thinning. Generally, they could be classified into three main categories based on the preserved degrees of subdermal structures: thin [13] or ultrathin [14] flap elevated at the suprafascial plane; superthin flap [15] with minimal adipose tissue for subdermal plexus preservation; and pure skin perforator flap [11,12], which is thinned until the dermis without the subdermal plexus (Figure 1a).

### Current imaging guidance for harvesting a thinned perforator flap

Ideal imaging guidance for flap thinning needs to fulfil the following criteria. (1) Provide both anatomical and haemodynamic real-time flap information. Specifically, subcutaneous perforator branches should be clearly outlined. Considering the sensitivity of flap haemodynamics towards tension [16], constantly alternating perfusion should be monitored throughout surgery for timely intervention before progression to unsalvageable flap necrosis. (2) It is accurate, reliable, reproducible, safe, noninvasive, economical, broadly available and stable.

Ongoing efforts are directed towards developing the ideal imaging guidance to best fulfil the abovementioned criteria. Existing imaging modalities for thinned perforator flap harvest guidance are discussed below from two perspectives: (1) anatomic delineation of the subcutaneous perforator branches and (2) perfusion assessment of the thinned perforator flap.

#### Anatomic delineation of perforator branches in the subcutaneous tissue

For maximal protection of the essential subcutaneous vasculature, approaches including microdissection [4–6], superficial fascia plane elevation [13], honeycomb technique with ultrasonic aspirator [17], secondary thinning, setting up safety limits on flap dimension and thickness [18] and 0.5–3 cm [3,4,13,19] cuff preservation around the perforator, have been attempted. Therefore, in addition to perforator emergence identification at the muscle fascia, imaging for thinned flaps necessitates features that capitalize on the subcutaneous perforator course and haemodynamics simultaneously, allowing surgeons to go beyond these approaches to produce better and safer designs of thinned perforator flaps.

##### Computed tomography angiography

Computed tomography angiography (CTA) integrates computer-analysed X-ray images and contrast medium to produce high-resolution reconstructions of vasculature imaging. CTA remains the gold standard for perforator mapping due to its capability to provide a comprehensive 3D perforator’s ‘road-map’ from its source vessel.

CTA has also long been used in cadaveric studies for both anatomical and haemodynamic assessments of perforators up to the subdermal plane [2,10,20]. With technological sophistication, CTA is currently equipped with multidetector-row computed tomography that contributes to enhanced spatial resolution and simultaneous reduction of radiation dose, thus allowing precise demonstration of the location, calibre and branching pattern of subcutaneous perforator branches as small as 0.3–0.5 mm [21]. The post-processing 3D volume rendering technique allowed surgery simulation through illustration of their subcutaneous course along with their relationships with surrounding structures, preparing the surgeons for any anatomical variation encountered [22].

Fang *et al*. [23] reported excellent sensitivity and a specificity of 100% for CTA in thinned deep inferior epigastric artery perforator flap harvest for perineal reconstruction. Rather than the previously suggested Scarpa’s fascia, superficial inferior epigastric vein, which is the easily visualized main venous return vessel via CTA, should be adopted as the superficial limit to enable further flap thinning [24]. CTA is also beneficial in flap thickness customization, especially in obese individuals [25].

However, a high dose of contrast medium is required for delicate superficial branch visualization [26], posing risks in patients with renal insufficiency. CTA is also inappropriate for continuous flap monitoring and is too bulky for intraoperative use. Although CTA has been well exploited for dynamic vascularity assessment in cadavers [20], the current clinical use of CTA is limited to static imaging and lacks haemodynamic information. Perhaps, its potential in demonstrating a complete picture of perforator flaps that incorporate both anatomic and physiological aspects still awaits unleashing.

##### Colour Doppler ultrasound or colour-coded duplex sonography

By combining both B-mode and colour Doppler imaging, colour Doppler ultrasound (CDU) provides 2D colour-coded images of the vasculature, along with their corresponding blood flow direction and velocity.

Since the first use of CDU by Hallock [27] in flap surgery, there has been tremendous progress due to its superior delineation of the superficial microcirculation. Its use in flap thinning guidance was mentioned early in 2008 for course pattern investigation of the thoracodorsal artery perforator [28]. What makes CDU stand out is its ability to provide both anatomical and haemodynamics information on the perforator. However, most studies have only focused on its utility in perforator architecture demonstration. In fact, perforator haemodynamics should not be overlooked, as evaluation of the pulsatility index and peak systolic velocity provide invaluable hints on the best-matched perforator to the recipient vessels for optimum flap perfusion [29].

Its noninvasive and radiation-free nature, affordability, portability and capability of providing additional key haemodynamics information have facilitated its application in both pre- and intra-operative settings, which make it potentially more advantageous than the gold standard CTA. However, its intraoperative usage specifically for thinned flap harvesting has been sparsely reported. The high accuracy and sensitivity in perforator localizations of CDU [30] have contributed to its reliability as a preoperative flap evaluation device in thin anterolateral thigh (ALT) flaps [31]. What is more interesting is the recent successful direct pure skin and superthin perforator flap elevations under CDU guidance [32]. Precise perforator recognition by CDU-permeated distal-to-proximal dissected ‘perforator branch flap’ [29] avoids additional thinning as with routine proximal-to-distal dissection [32].

The capability of CDU in identifying capillary perforators of ~0.3 mm in diameter [33] has been reported, yet Hallock [27] mentioned the unreliability of CDU in perforators <0.5 mm in diameter, which is undeniably true from our experience [34]. Current CDU technology only produces 2D images that lack detailed whole structural information. Real-time haemodynamics information obtained may be subjective and cannot be reproduced for independent review. As shown in our previous study, this problem may be alleviated with the help of the ‘magnifying ability’ of contrast-enhanced ultrasound (CEUS) with 3D reconstruction that is based on sulfur hexafluoride microbubbles for enhancements in blood echo and signal-to-noise ratio [34]. Perforators as small as 0.5 mm that appeared dotted and noncontinuous with CDU could be detected and ‘amplified’ by CEUS, aiding in the identification of delicate yet reliable perforator branches, especially those that are more superficially located in the subdermal plane [34].

##### Ultra-high frequency ultrasound (UHF-US)

Conventional ultrasound devices with a lower frequency of linear probes might not be sufficient for detailed imaging of superficial perforator branches.

Recently, UHF-US with a distinctly increased frequency of up to 70 MHz has permitted the demonstration of superficial structures located within 10.0 mm of the surface at a resolution of 30 μm [35]. The superiority of UHF-US for assessing superficial structures has made it an invaluable device for assessments of dermatological conditions [35], peripheral vasculature, cutaneous nerves [36] and recently thinned perforator flaps [19,37].

Preoperative illustration of the subcutaneous perforator course has allowed surgeons to have the flap elevation plane planned, thereby enabling direct elevation of the thinned perforator flap without the concern of anatomical variation during the dissection and elevation procedure [19]. Extreme visualization of the perforator branches from the dermis entrance up to the intradermal plexus using 48 and 70 MHz probes has resulted in successful harvest of thin, superthin and pure skin perforator flaps [19,37]. Of course, due to its portability, its use could be extended to include further defatting upon flap elevation in the intraoperative setting, although this has not been reported before.

However, its use is limited to the visualization of very superficial structures. For a comprehensive picture of the perforator, conventional ultrasound at 18 MHz is still required beforehand for visualizing its emergence in the muscular plane [19,37]. In fact, the use of high-frequency CDU (5–17 MHz) was proven to be adequate for visualizing the emergence and branching pattern of superficial vasculature of as small as 0.2 mm in diameter in the reverse digital artery island flap of the ulnar side of the thumb [38]. In addition, UHF-US is currently still very costly and not widely available. Although it may seem fascinating, questions regarding the practical significance of such detailed demonstration of the intradermal plexus up to its layering [36] in thinned flap harvesting remain to be investigated.

##### Photoacoustic tomography imaging

Photoacoustic tomography imaging (PAT), an emerging hybrid vascular imaging system, incorporates the benefits of optical illumination and ultrasound detection. Target tissues absorb light energy irradiated by a near-infrared (NIR) pulse laser and emit ultrasonic waves, minimizing the optical scattering effect for enhanced-resolution imaging [39]. Endogenous biological contrast substances such as oxy- and deoxy-haemoglobin, melanin and lipids, provide structural and functional information on the tissues. Since the first reported use of PAT in the imaging of brain tumours [40], it has drawn much attention and has been used in areas as diverse as oncology, dermatology, cardiology and neurology [39].

Recently, PAT has been reported to have high accuracy in the 3D delineation of subcutaneous perforator branches of the ALT flap [26,41]. Its colour-coded demonstration of vasculature depth permits straightforward recognition of perforator branches according to flap thicknesses [26,41].

However, an effective intraoperative navigation system is currently unavailable. In addition to structural delineation of vasculature, considering its functional imaging use in detecting haemodynamic changes through haemoglobin oxygenation assessments in other applications [39], perhaps its potential in monitoring flap thinning-elicited haemodynamic changes has yet to be uncovered. As the use of PAT in the field of plastic surgery is very much in its infancy, its significance in thinned flap harvest has yet to be thoroughly explored.

#### Perfusion assessment of the thinned perforator flap

Perfusion-related flap necrosis remains the commonly encountered complication of perforator flaps, especially in thinned flaps. Attributable to limitations arising from cost and availability, surgeons primarily rely on their experiences in perfusion evaluation through observation of flap skin colour, turgidity, temperature, capillary refill time and dermal bleeding. Additional measures with adjuvant intraoperative imaging that facilitate accurate prediction of flap viability through real-time quantitative assessment of flap perfusion could provide trustworthy guidance on effective intervention for favourable surgical outcomes.

##### Indocyanine green angiography

Indocyanine green (ICG) is a water-soluble, sterile and FDA-approved NIR tricarbocyanine fluorescent dye that has been used for functional evaluation of the heart and liver, ophthalmic angiography, tumour resection guidance [42] and perforator flap assessment [17]. ICG possesses a spectral absorption range and peak emission of 750–800 nm and 832 nm, respectively [42]. Once injected, given its strong binding ability to plasma proteins, visualization of dye distribution with an 806 nm NIR laser allows a penetration depth of up to 2 cm [43], corresponding to the fascial level. One additional feature that makes indocyanine green angiography (ICGA) stand out is that it allows synchronized real-time perforator mapping and quantitative flap perfusion evaluation [17]. Such features can be invaluable, especially when larger flaps are involved [44]. A recent report on a successful unprecedented full-thickness skin graft-like thinned perforator flap with guidance further ascertained its significance in thinned flap harvesting [12].

Its portability and repeatability have made it a popular device for intraoperative perforator and microsurgical anastomosis assessments [45], preventing avoidable vascular-related flap complications. Compared to the gold standard CTA, ICGA seems more beneficial in delineating delicate perforator branches up to the intradermal level of <0.2 mm diameter [12]. Tissue expansion, a popular approach to create perforator flaps with decreased adipose and dermal thicknesses that simultaneously match the colour and texture of recipient sites [46], tends to evoke haemodynamic changes through choke anastomosis opening and neovascularization [47]. ICGA is beneficial in perforator localization of such thin flaps of <8 mm thickness and with greatly augmented vascularity [48,49].

In addition, unlike many of the other commonly used angiographies, ICGA could provide a real-time haemodynamic overview of a specific zone rather than of a single vessel, which would be more reasonable for perioperative flap monitoring. As flap perfusion is constantly alternating until its final transposition to the donor site, real-time flap perfusion assessment is crucial throughout surgery, especially when extreme thinning or a large thinned flap is planned. Intraoperative flap perfusion evaluation using ICGA has been proven with a high accuracy of 98.6% and sensitivity of 90.9% in a recent meta-analysis [50]. Thus, apart from direct thin flap elevation, this feature of ICGA has also contributed to its feasibility in flap defatting after elevation where the degree of flap thinning can still be continually adjusted according to perfusion mapping during the procedure [17]. Its application could also be further broadened to include flaps where vascularization has been augmented through prefabrication, supercharging or tissue expansion, to assist in tailoring optimal flap designs based on the perfusion territories of the pedicle [49]. The efficacy of ICGA as a haemodynamic analysis tool for quantitative measurements of arterial inflow and venous outflow, has been further proven by our previous work on challenging cervicofacial reconstruction with pre-expanded multipedicled perforator flap with augmented blood supply, prefabricated and/or supercharging, which demands an even thinner and larger flap yet with secured vascularization [51]. Reliable flap necrosis prediction has helped to avoid unfortunate vascular-related flap outcomes particularly in the distal end due to in-time flap salvage [12,49]. Moreover, ICGA could even be used for expansion capsule pressure optimization during the expansion period, helping to eradicate expansion-related complications, as demonstrated in our previous work [52].

**Figure 2.**
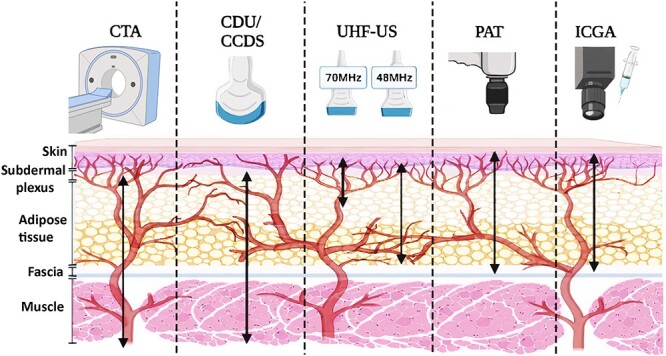
Demonstration of the imaging depth limit of imaging modalities that have been used to harvest thinned perforator flaps. Created with BioRender.com. *CTA* computed tomography angiography, *CDU* colour Doppler ultrasound, *CCDS* colour-coded duplex sonography, *UHF-US* ultra-high frequency ultrasound, *PAT* photoacoustic tomography, *ICGA* indocyanine green angiography

However, ICGA is not cost-effective and is undesirable in patients with liver problems, severe allergies or pregnancy. With its limited image capture area, ICGA is problematic in perfusion assessments of large flaps [45]. ICGA is prone to underestimation of flap survival by 6–10% [53], causing overresection of viable tissue or unnecessary surgical intervention. There is still ongoing research in defining the threshold for flap necrosis [54]. Noncontinuous data provided by ICGA with low photostability lack reliability [55]. Despite its widespread use in flap perfusion assessment [12,17], studies directed towards its specific application in vasculature delineation in thinned perforator flaps are comparatively scarce. A brief overview of imaging modalities that have been used to harvest thinned perforator flaps, along with their imaging depth limits, are summarized in Table 1 and Figure 2. See Table S1 in the online supplementary material for detailed information on their uses in the previous literature.

**Table 1:** Overview of imaging modalities that have been used to harvest thinned perforator flaps

**Imaging modalities**		**CTA**	**CDU/CCDS**	**UHF-US**	**PAT**	**ICGA**
**Imaging depth limit**	**Superficial**	Vessels up to 0.3–0.5 mm in diameter [21]	Entrance to the dermis Vessels up to 0.3–0.5 mm in diameter [27,33]	Up to the intradermal plexus [35] Microanatomical structures up to 30 μm [19]	Uppermost layer of epidermis	Intradermal level Vessels ≤0.2 mm in diameter [12]
	**Deep**	Interseptal or intramuscular course	Interseptal or intramuscular course	10 mm [35] (with frequency of 70 MHz) 23.5 mm [35] (with frequency of 48 MHz)	13 mm [41] (Deep fascia)	2 cm [43]
**Parameters**		Location, course, branching pattern and calibre of perforators	Vessel calibre Arborization pattern of perforator in adipose layer Pulsatility index (PI) Peak systolic velocity (PSV) Resistance index (RI) End diastolic velocity (EDV)	Depth of superficial and Scarpa’s fascia Arborization pattern of perforator in adipose layer	Branching pattern of perforator Depth of vessel (colour scale)	Perforator mapping Perfusion pattern Starting intensity Ingress rate Curve integral End intensity Egress rate
**Advantages**		3D visualization of perforators Operator independent	Non-invasive Portability Both anatomic and haemodynamic data of perforators	Real-time Non-invasive	Non-invasive 3D visualization of branching vessels	Real-time Synchronized assessment of flap perfusion and perforator mapping Reproducible Perforator detection in thinned flap <8 mm [48]
**Disadvantages**		High dose of contrast medium for visualization of superficial vasculature Radiation Lack portability Affected by metallic artefacts, haematoma or insufficient fat	Operator dependent Time-consuming for detailed imaging 2D imaging	High cost Not widely available Limited to visualization of very superficial structures Operator dependent	Difficulty in differentiating arteries and veins [26] Difficulty in visualizing vertically oriented vessels [26,41] Lack of intraoperative navigation system Sensitive to motion	High cost Invasive Non-continuous Limited imaging area Underestimation of flap survival by 6–10% [53] Poor photostability [55]

*CTA* computed tomography angiography, *CDU* colour Doppler ultrasound, *CCDS* colour-coded duplex sonography, *UHF-US* ultra-high frequency ultrasound, *PAT* photoacoustic tomography, *ICGA* indocyanine green angiography

To the best of our knowledge, only ICGA has been reported as an intraoperative guidance device in thinned flap harvesting thus far. As long as dye injection is not of great concern, ICGA seems to remain the preferred choice, as it provides synchronized anatomical and perfusion data, which are of particular importance in ensuring a successful reconstructive outcome. If dye allergy, cost and availability are of importance, there are alternative options available for flap perfusion assessment, which will be briefly discussed below.

##### Laser speckle contrast imaging

Through the detection of erythrocyte flow at a tissue depth of 300 μm [56], superficial dermal perfusion could be noninvasively quantified through speckle pattern change detection. In contrast to ICGA, laser speckle contrast imaging (LSCI) allows wide-field imaging of 24 × 24 cm for continuous perfusion monitoring of a large flap without repetitive dye injection [57]. Its efficacy has been reported in intraoperative cutaneous perfusion imaging of thin flaps, including full-thickness eyelid [16] and ALT flaps [58], and thus might be appropriate for thinned perforator flap perfusion assessment.

##### Dynamic infrared thermography

Thermal challenge-induced rewarming rate and pattern (temperature) changes in the area of interest permit indirect real-time subcutaneous tissue perfusion assessment. Heat signals emitted by perforators enable the ‘hot-zone/cold-zone’ concept of flap harvest, with care taken in the hot zone (perforator zone) for safe thinning [59]. In addition, the ability of dynamic infrared thermography (DIRT) to identify linking vessels between perforasomes through analysis of differential rewarming rates between perforators makes it an excellent option in large thinned perforator flaps that involve multiple perforators [60]. DIRT has recently gained popularity with the emergence of convenient smartphone-based thermographic cameras [61], which would be helpful in emergency and intraoperative settings.

##### Non-invasive tissue oximetry

Through flap tissue oxygen saturation (StO_2_) evaluation using NIR spectroscopy with penetration depths ranging from 0–10 mm [62], adipocutaneous branch injury-induced tissue hypoxia could be detected before noticeable clinical observation, significantly improving the flap salvage rate from 57.7 to 93.75% in microsurgical breast reconstruction [63]. The regional oxygen saturation index (OSI) is markedly correlated with the intraoperative ICG areas, suggesting its role as an alternative noninvasive intraoperative guidance tool, with an OSI of <0.75 [64], StO_2_ < 30% and drop rate of >20% per hour lasting for >30 min [65] being the threshold for the requirement for vascular intervention.

#### Suggested imaging algorithm for thinned perforator flap harvest

During the preoperative stage, the evaluation of perforator, including its localization and haemodynamics (vessel calibre and velocity), are of particular importance in ensuring safe design and planning. Due to spatial resolution limitations and inability to provide haemodynamic information, CTA, despite being the current gold standard for perforator mapping, seems insufficient for necessary subcutaneous branch information, especially in an inherently thin donor site. Yet, it is still mandatory for visualizing a 3D comprehensive course of perforator emergence in the muscular plane. It also lacks the ability to provide deterministic haemodynamic information. A practically advisable strategy may be to couple the use of CTA with CDU for additional crucial subcutaneous perforator course and hemodynamics information, or with CEUS or UHF-US in cases requiring visualization of more superficially located perforators of <0.5 mm, if available.

The intraoperative stage, on the other hand, emphasizes thorough perfusion assessment and delineation of subdermal or intradermal perforator branches throughout the flap elevation and debulking process. Regardless of the approach taken for thinned flap harvest, direct thin flap elevation or flap defatting after elevation, ICG remains the most desirable option for its provision of influential synchronized delineation of superficial perforator branches and perfusion information. Again, considerations regarding dye allergy, costs and availability to surgeons should be taken into account. In this case, a rational approach would be to combine the use of perfusion assessment imaging such as LSCI, DIRT or tissue oximetry, with CDU, CEUS or UHF-US for perforator branch delineation. The suggested algorithm for imaging of thinned perforator flap harvest is summarized in Table 2.

**Table 2:** Suggested algorithm for imaging thinned perforator flap harvest

	**Purposes**	**First option**	**Alternatives**
**Preoperative**	Perforator localization	CTA (gold standard)	DIRT^a^, CDU^a^, ICGA^a^
	Perforator haemodynamics (calibre, velocity)	CDU	UHF-US^b^, CEUS^b^
**Intraoperative**	Flap perfusion	ICGA	LSCI^a^, DIRT^a^, Tissue oximetry^a^
	Superficial vasculature delineation	ICGA	CDU^c^, UHF-US^b^, CEUS^b^

^a^Not commonly used for this purpose

^b^Preferable if perforator is <0.5 mm in diameter

^c^Preferable if perforator is >0.5 mm in diameter

*CTA* computed tomography angiography, *DIRT* dynamic infrared thermography, *CDU* colour doppler ultrasound, *ICGA* indocyanine green angiography, *UHF-US* ultra-high frequency ultrasound, *CEUS* contrast-enhanced ultrasound, *LSCI* laser speckle contrast imaging

### Future perspectives

As there is no single ideal imaging modality for thinned perforator flaps, there has been a continuing need for the development of improved imaging devices. In addition, it is often still left to a surgeons’ imaginations to fuse many images to create accurate representations of patients. In the future, we look forward to seeing breakthroughs in efforts to resolve the abovementioned issues through innovations in imaging agents and devices and a digital surgical environment that incorporates mixed reality (MR) and artificial intelligence (AI) technologies.

ICGA, the current desirable option for flap thinning guidance, possesses major drawbacks including limited photostability and fluorescence quantum yields [55,66]. Feng *et al*. [55] proposed the use of lead sulfide (PbS) quantum dots (QDs) as an imaging agent for flap perfusion assessment. QDs possess excellent photostability, low cytotoxicity and enhanced detection depth and resolution in the shortwave-infrared (SWIR) window of longer wavelengths (1000–2300 nm) [55,66]. Real-time and long-term *in vivo* SWIR fluorescence imaging based on PbS QDs permitted precise flap perfusion assessment in rat models [55]. More recently, Ibrahim *et al*. [67] introduced an intradermally injectable nonbiodegradable phosphorescence oxygen sensor made up of benzoporphyrin dye and pHEMA hydrogel that allows continuous, real-time and optical monitoring of flap perfusion that lasts from months to a year.

Both of the abovementioned studies are currently still in the animal experimental stage and await further studies for translation into clinical practice. Regardless of how advanced the imaging modality being developed, there is no way of guaranteeing optimal flap design and elevation without transferring the information obtained from the imaging to the surgical field intraoperatively for direct guidance. There is no doubt that MR (virtual plus augmented reality) and AI are currently the two major players in the technological world. Thinned flap harvest, with a high demand for precision and accuracy, would benefit from the combined usage of existing imaging modalities with MR and AI. For instance, the first commercially available MR device, Microsoft Hololens, permits real-time superimposition of a complex yet critical 3D vascular map obtained through preoperative CTA onto surgical sites for intraoperative navigation [68]. By enabling the surgeons to ‘see-through’ tissues, perforator branches, particularly those that are superficially placed, could be accurately identified and constantly seen throughout the procedure, resulting in decreased overall operative duration, better customized flap design and thickness and avoiding unnecessary painstaking dissection and vasculature injury. With future integration of haemodynamic assessment devices into MR, we expect to see further reliability enhancement in this flap guidance system. Medicine, in just a few decades, has leaped forward with evidence-based practice. Machine learning, a subfield of AI, seems to be the best approach for processing the vast amount of data collected. Acknowledging its efficiency in donor site-related complication prediction [69], automatic perforator detection [70] and postoperative monitoring [71] in microsurgical reconstruction, we foresee the possibility of machine learning enhancing the success rate in thinned perforator flaps through the abovementioned perspectives.

## Conclusions

Over the past decades, numerous new technologies have been developed to surpass the limitations imposed by conventional imaging. However, to date, the lack of high-quality evidence supporting their efficacies has impeded the establishment of comprehensive clinical guidelines on the use of imaging in thinned perforator flap harvest. In the future, it can be expected that MR and AI approaches will be implemented for more favourable reconstructive outcomes.

## Abbreviations

AI: Artificial intelligence; ALT: anterolateral thigh; CDU: Colour Doppler ultrasound; CEUS: Contrast-enhanced ultrasound; CTA: Computed tomography angiography; DIRT: Dynamic infrared thermography; ICGA: Indocyanine green angiography; LSCI: Laser speckle contrast imaging; MR, mixed reality; NIR, Near-infrared; OSI, Oxygen saturation index; PAT: Photoacoustic tomography imaging; QD, Quantum dot; StO_2_: Oxygen saturation; SWIR, Shortwave-infrared; UHF-US: Ultra-high frequency ultrasound

## Funding

This work was supported by grants from National Natural Science Foundation of China (81 772 086 and 82 072 177), ‘Two Hundred Talent’ program, ‘Outstanding Youth Medical Talents’ Shanghai ‘Rising Stars of Medical Talent’ Youth Development Program and Shanghai Jiao Tong University ‘Chenxing’ Youth Development Program (Associate Professor Type A).

## Authors’ contributions

Conception and design: YMK, XH, TZ; collection and assembly of data: YMK; data analysis and interpretation: YMK, XH, SCG; graphic illustration: YMK; manuscript writing: all authors; manuscript revision: YMK, XH, SCG; final approval of manuscript: all authors.

## Conflict of interest

None declared.

## Supplementary Material

Supplementary_Table_1_tkab042
