# Supplementary material for: Imaging for thinned perforator flap harvest: current status and future perspectives
Source: Burns Trauma. 2021 Dec 17;9:tkab042. doi: 10.1093/burnst/tkab042 (PMC8677592; doi:10.1093/burnst/tkab042)
Supplement: Supplementary_Table_1_tkab042 [file supplementary_table_1_tkab042.docx]

**Supplementary Table 1.** Review of the literature on imaging modalities used in harvesting thinned perforator flaps.

| **References** | **Imaging modality** | **Usage**  **(Pre-op/Intra-op)** | **Approaches of thinned flap harvest** | **Reliability of imaging modality** | **Final flap sizes and thicknesses** | **Reported outcomes**  **(Flap survival and vascular-related complications)** | **Level of evidence** |
| --- | --- | --- | --- | --- | --- | --- | --- |
| Heredero et al., 2020[25] | CTA | Pre-op | Superficial fascia elevation + thinning (customization) | Not mentioned | Size: 5×9 cm  Thickness: 0.4–3 cm | - 9 flaps survived - 1 failed due to vasospasm | IV |
| Fang et al., 2011[23] | CTA | Pre-op | Flaps were thinned in the plane inferior to the superficial inferior epigastric vein | 100% sensitivity, 100% specificity in anatomical delineation of DIEP flap | Size: 18×5 to 24×8.5 cm  Thickness: 6–11 mm | All 12 flaps survived:   - Partial necrosis in one flap measured 24×8.5 cm; | IV |
| Imaizumi et al., 2020[29] | CDU/CCDS | Pre-op | Simultaneous perforator dissection and primary thinning | Accurate delineation of perforator branches in all cases | Size: 10×4.5 to 30×20 cm  Thickness: 4–18 mm | All of the 30 flaps survived:   - Partial necrosis in 1 flap - Epidermolysis in 2 flaps - Flap congestion in 1 flap - 5 flaps required further secondary debulking | II |
| Yamamoto et al., 2021[32] | CDU/CCDS | Pre-op | Subdermal elevation (dermis as dissection plane); distal-to-proximal perforator dissection | Precise localization of pure skin perforator into dermis | Size: 3.5×2 to 27×8 cm  Thickness: 1.0–4.0 mm | All of the 36 pure skin perforator or superthin flaps survived:   - Partial necrosis in 2 flaps (healed with conservative treatment) | IV |
| Visconti et al., 2020[19] | UHF-US | Pre-op | Direct thinned flap elevation (elevation plane depends on branching pattern of perforator) | 100% agreement with intraoperative findings | Size: 5×8 to 7.5×23 cm  Thin and superthin flap (exact thickness not mentioned) | All of the 7 flaps survived with satisfactory aesthetic and functional outcomes | IV |
| Yoshimatsu et al., 2019[37] | UHF-US | Pre-op | Superficial fascia elevation + further thinning | Accurate localization of the dermis entry point of vessels in all cases | Size: 8×3 to 12×5 cm  Thickness: 0.6–0.9 mm | All of the 6 flaps survived with no complications | IV |
| Tsuge et al., 2020[26] | PAT | Pre-op | Conventional anterolateral thigh flap elevation in 8 flaps, with only 2 undergoing further thinning (details not mentioned) | PAT imaging matched the intraoperative findings within 10 mm in all cases; difficulty in visualizing blood vessels in vertical direction | Size: 12×6 to 33×10 cm  Thickness: Flap thinning was only performed on 2 flaps (Final thicknesses: both 5.0 mm) | - All of the 8 flaps survived with no complications | IV |
| Fan et al., 2018[17] | ICGA | Intra-op | Comparative study between MDT and HCT in flap thinning | ICGA for perfusion assessment: ≤30% as an indication of  tissue ischaemia and excised before flap thinning or final pedicle ligation | HCT group  -Size: 6.5×4 to 20×14.5 cm  -Thickness: 4.3±0.4 mm  MDT group  -Size:7.5×4.5 to 18.5×10.5 cm  -Thickness: 6.8±0.7 mm | All of the 40 flaps in both groups survived with no complications. | II |
| Narushima et al., 2018[12] | ICGA | Intra-op | Pure skin perforator flap elevation with 11 flaps undergoing excess skin and fat removal around vascular pedicles | ICGA to study flap perfusion pattern: radial diffusion pattern, direct linking vessels in intradermal  layer and intradermal arteriovenous shunts | Size: 39 ± 22 cm^2^  (range: 3–90 cm^2^)  Thickness: 2.1 ± 0.2 mm (range: 2–3 mm) | All of the 40 pure skin perforator flaps survived:   - Flap congestion in 1 flap on Day 2 postop (venous re-anastomosis required) - Partial necrosis in 1 case - Epidermal necrosis in 1 case | IV |
| Han et al., 2020[49] | ICGA | Intra-op  (expander placement and flap  elevation) | Pre-expanded perforator flap (brachial artery perforator flap) | ICGA for perforator localization and confirmation of flap perfusion:   - Accurate localization in all cases | Size: 15×6 to 27×14 cm^2^  Thickness: 4–20 mm | All of the 16 flaps survived with no complications. | IV |

CTA, Computed tomography angiography; CDU, Colour Doppler ultrasound; CCDS, Colour-coded duplex sonography; UHF-US, Ultrahigh frequency ultrasound; PAT, Photoacoustic tomography; ICGA, Indocyanine green angiography; HCT, Honeycomb technique; MDT, Microdissection technique
